# INTRODUCTION OF THE NEW LYMPHOPARIETAL INDEX FOR GASTRIC CANCER PATIENTS

**DOI:** 10.1590/0102-672020190001e1441

**Published:** 2019-08-26

**Authors:** Manuel FIGUEROA-GIRALT, Attila CSENDES, Katya CARRILLO, Stefan DANILLA, Enrique LANZARINI, Italo BRAGHETTO, Maher MUSLEH, Solange CORTÉS

**Affiliations:** 1 Department of Surgery, Universidad de Chile Clinical Hospital, Santiago, Chile.

**Keywords:** Gastric cancer, Survival, Prognosis, Index, Câncer gástrico, Survival, Prognosis, Index

## Abstract

*****Background***:**

The identification of prognostic factors of gastric cancer (GC) has allowed to predict the evolution of patients.

*****Aim***:**

Assess the reliability of the lymphoparietal index in the prediction of long-term survival in GC treated with curative intent.

***Method*:**

Prospective study of the Universidad de Chile Clinical Hospital, between May 2004 and May 2012. Included all gastric cancer surgeries with curative intent. Exclusion criteria were: gastrectomies due to benign lesions, stage 4 cancers, R1 resections, palliative procedures, complete esophagogastrectomies and emergency surgeries.

***Results*:**

A total of 284 patients were included; of the sample 65.4% were male,mean age of 64.5 years,75% were advanced cancers, 72.5% required a total gastrectomy, 30 lymph nodes harvest. Surgical morbidity and mortality were 17.2% and 1.7%. 5-year survival was 56.9%. The N+/T index could predict long-term survival in all de subgrups (p<0.0001), although had a reliable prediction in early GC (p=0.005), advanced GC (p<0.0001), signet ring cell GC (p<0.0001), proximal GC (p<0.0001) and distal GC (p<0.0001). The ROC curves N+/T index, LNR and T classification presented areas below the curve of 0.789, 0.786 and 0,790 respectively, without a significant statistical difference (p=0.96).

*****Conclusion***:**

The N+/T index is a reliable quotient in the prognostic evaluation of gastric adenocarcinoma patients who have been resected with curative intent.

## RESUMO

*****Racional***:**

A identificação de fatores prognósticos do câncer gástrico (GC) permitiu predizer a evolução dos pacientes.

***Objetivo*:**

Avaliar a confiabilidade do índice linfoparietal na predição de sobrevida em longo prazo em pacientes tratados com intenção curativa.

***Método*:**

Estudo prospectivo do Hospital das Clínicas da Universidade de Chile, entre maio de 2004 e maio de 2012. Todas as operações de câncer gástrico foram com intenção curativa. Os critérios de exclusão foram: gastrectomia por lesões benignas, cânceres estágio 4, ressecções R1, procedimentos paliativos, esofagogastrectomias completas e operações de emergência.

Foi incluído um total de 284 pacientes; da amostra 65,4% eram homens, com média de idade de 64,5 anos, 75% eram cânceres avançados, 72,5% necessitaram de gastrectomia total e 30 coletas de linfonodos. A morbimortalidade cirúrgica foi de 17,2% e 1,7%. Sobrevida em cinco anos foi de 56,9%. O índice N +/T pôde predizer a sobrevida em longo prazo em todos os subgrupos (p<0,0001), embora tivesse previsão confiável em GC precoce (p=0,005), GC avançado (p<0,0001), célula GC de anel de sinete (p< 0,0001), GC proximal (p<0,0001) e GC distal (p<0,0001). As curvas ROC N +/T, LNR e T apresentaram áreas abaixo da curva de 0,789, 0,786 e 0,790, respectivamente, sem diferença estatística significativa (p=0,96***).***

***Conclusão*:**

O índice N +/T é um quociente confiável no prognóstico na avaliação de pacientes com adenocarcinoma gástrico que foram ressecados com intenção curativa.

## INTRODUCTION

Over the last 40 years, gastric cancer (GC) has experienced significant changes with regards to treatment and prognosis. Thus, an increase in patients with undifferentiated cancers (particularly signet cell cancer), tumors in the superior third of the stomach,and a greater proportion of total gastrectomies have been observed, in addition to a significant decrease in surgical mortality and an improvement in a 5-year global survival rate(SVg5)^2^ ^,^ ^4^ ^,^ ^7^ ^,^ ^17^. 

Identification of some prognostic factors has contributed in the therapeutic decision making process^15^ ^,^ ^18^. In Chilean reality, there are few studies with multivariate analysis^3^ ^,^ ^10^ ^,^ ^20^. Recently a new prognostic factor was created and validated, the lymphoparietal index (N+/T)^10^.

The main objective of this study was to assessthe reliability of the lymphoparietal index in the prediction of long-term survival in GC treated with curative intent.

## METHOD

Prospective analysis of the oncological database of a Chilean University Hospital (Universidad de Chile Clinical Hospital) between May 2004 and May 2012.This article does not contain any experimental studies with human or animal subjects performed by any of the authors.

### Patients 

All patients with gastric adenocarcinoma and Siewert II and III esophagogastric junction in the adult population, surgically treated with a curative intent, were included. Subtotal, total and extendedgastrectomies were included. 

Exclusion criteria included: gastrectomies due to benign lesions, stage 4 cancers according to the 7^th^ edition TNM classification, R1 resections, palliative procedures, complete esophagogastrectomiesand emergency surgeries.

### Surgical technique

Defined in previous report^10^

### Definitions

Were used: 1) TNM classification was standarized using the AJCC 7^th^ edition^15^ (Table 1); 2) the lymphoparietal index (N+/T) calculates the quotientbetween the number of lymph nodes that are positive for adenocarcinoma metastasis and the T classification of the patient, andfor mathematical purposes, the T1a and T1b subdivisions were indistinctly acceptedas 1 in the denominator (examples: 1/T1a = 1/1 = 1, 6/T3 = 6/3 = 2, 24/T4 = 24/4 = 6); the ratio results were divided into N+/T A (0), N+/T B (0.1-3) and N+/T C (>3) subgroups; 3) the lymph node index (LNR) was divided into four groups: group 0: 0% of lymph nodes compromised, group 1: from 1% to 9% of lymph nodes compromised, group 2: from 10 to 25% of lymph nodes compromised and group 3: more than 25% of lymph nodes compromised^1^; 4) surgical mortality was defined as occurring from the moment of surgery up to postoperative day 30.

**TABLE 1:** TNM classification (n=284)

	n	%
Classification T		
T1	71	25
T2	31	10.9
T3	73	25.7
T4	109	38.3
Classification N		
N0	114	40.1
N1	33	11.6
N2	39	13.7
N3a	55	19.3
N3b	43	15.1
Stage		
IA	67	23%
IB	14	4.9%
IIA	27	9.5%
IIB	34	11.9%
IIIA	31	10.9%
IIIB	51	17.9%
IIIC	60	21.1%

Classification TNMacording to 7ª ed. of 2010 AJCC

### Follow-up 

The present study had 100% follow up. The database was completed in a prospective manner: the survival update was carried out annually using the database of our hospital and the Chilean Civil Registry

### Statistical analysis

The prognostics factors evaluated were demographic, clinical, surgical, anatomopathological and prognostic indexes. The distribution of variables was determined by the Shapiro-Wilk test*.* In accordance with this test, the continuous variables with parametric distribution (ordinal) were expressed on average and with a standard deviation (SD), while for the continuous variables with non parametric distribution (nominal) the median and inter-quartile (IC_25%-75%_) ranges were used. The categorical variables were described in percentages. The Fisher, x^2^, t student and Wilcoxon Rank-Sum tests were used based on the characteristics and distribution of the variables. For the analytical statistical analysis, the Stata^R^14 program was used and p<0.05 was considered statistically significant. Univariate and multivariate analyses were performed calculating the odds ratio (OR) with a 95% confidence interval (CI). The Kaplan-Meier method in the Prism7^MR^ program was used to calculate the survival curves. The Stata^R^ program was used to create the ROC curves.

## RESULTS

A total of 284 patients were included with a mean age of 64.5 years (+/-12.7 DS) of which 65.4% were male. 69.7% of all patients presented co-morbidities with high blood pressure, tobacco use and diabetes being the most common with 38.3%, 34.8% and 17.6 % respectively. 24.6% of all patients presented first-degree relatives with gastric cancer. 

In reference to the surgical technique, 72.5% of patients required a total gastrectomy, 19.7% a subtotal gastrectomy and 7.8% an extended gastrectomy. The organs additionally resected included the pancreas (41%), distal esophagus (27%), colon (14%), small intestine (14%) and kidney (4%). The lymphadenectomy was D1 in 14.7% and D2 in 85.2%. The mean global lymph node harvest was 30 lymph nodes (IC_25-75%_: 22-41). The number of lymph nodes harvested during D1 lymphadenectomies were 28 (IC_25%-75%_:19-32.5), while 33 were harvested in D2 lymphadenectomies (IC_25%-75%_: 24-44). Mean operating time was 208 min (+/-63.8 DS. All samples (100%) were R0 resections. 

The mean hospital stay of patients was nine days (CI_25-75%_: 7-11). Postoperative surgical morbidity corresponded to 17.2%, while surgical mortality consisted of five patients (attributed to three esophagojejunostomy filtrations, one pneumonia associated to mechanical ventilation and one duodenal stump fistula) which represented 1.7%.

The histopathological study revealed that 58.4% of tumors were of the intestinal type while 36.6% diffuse (undifferentiated or with signet ring cells) and 4.9% were mixed. 25% of the study corresponded to incipient cancers and 75% to advanced. Patients with signet ring cells represented 24.2% of the total sample. Lymphovascular invasion was identified in 50.3% of the sample, while perineural invasion was observed in 41.5%. The mean of compromised lymph nodes per person was 2(IC_25-75%_: 0-9). Table1 shows the TNM classification of patients. 

The mean global survival was 69.9 months (interval between 1-158 months, DS +/- 47.9). The rate of patients with a SVg5 was 56.9%. The SVg5 of these was 90.1% in incipient and 44.6% in advanced GC. 

**FIGURE 1:**
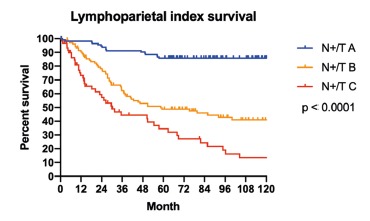
Survival according to N+/T index

**FIGURE 2:**
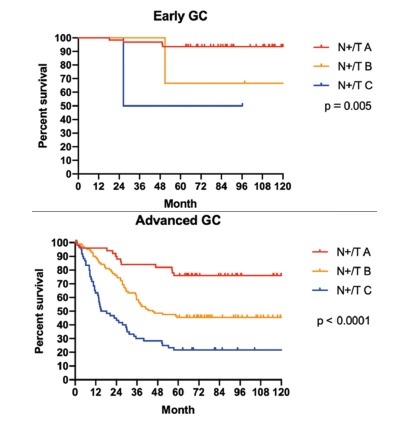
Survival according to N+/T index adjusted by early and advanced GC

**FIGURE 3:**
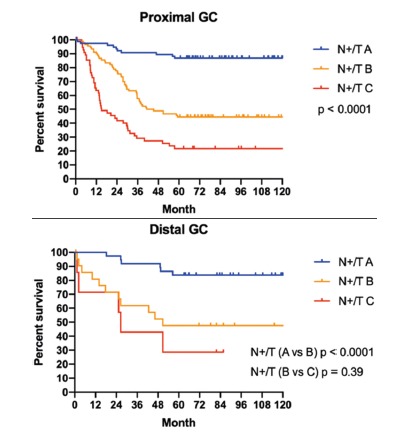
Survival according to N+/T index adjusted by proximal and distal GC: proximal GC included those in the fundus and body which received total gastrectomy and distal GC where the ones located in the antrum which received subtotal gastrectomy

**FIGURE 4:**
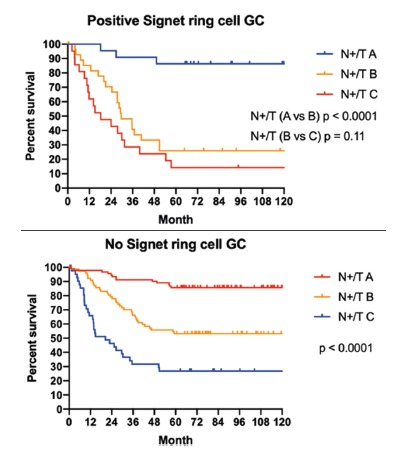
Survival according to N+/T index adjusted by signet ring cell condition of GC

Global survival according to the N+/T index in our population presented significant differences in each group (N+/TA, N+/TB and N+/TC) with p<0.0001 (Figure 1). Survival according to N+/T index adjusted to early/advanced GC, proximal/distal GC and signet ring cell GC, are shown in Figures 2, 3 and 4 respectively.

The ROC curve of the N+/T index and the comparative curves between the T and lymph node ratio predictive variables are represented in Figure 4. The area below the N+/T curve is 0.789 and the difference observed between the three variables in comparison did not reach a significant statistic, with p=0.96.

## DISCUSSION

The main results of this study suggest the following: 1) The oncological results comply with the quality models currently used for modern management of gastric cancer; 2) the N+/T index can be a very useful tool for predicting survival for more than five years in patients who underwent surgery for gastric adenocarcinoma with curative intent. 

The presentation and treatment of GC have changed over the past 40years. The frequency of distal tumors decreased from 64% to 25%, the intestinal type proportion: diffuse went from 3-4:1 to almost 1:1; the resectability rate has increased from 48% to 85% (p<0.001); 75% of all cases are total gastrectomies and surgical mortality has decreased from 25% to 0.8% (p<0.0001)^7^ ^,^ ^9^.

The epidemiological changes have different explanations^9^ ^,^ ^13^, while the best postoperative results are mainly due to two factors: “primary factor and secondary factor”. The first has to do with the role of the surgeon and the surgical team specialized in this pathology while the secondary factor is represented by the advances in medicine and support units such as intensive care, medical nutrition, physical therapies, interventional radiology, among others^6^. 

The patients studied in this article reflect part of these changes. This is the reason why practically 40% of cases are due to diffuse lesions, 2/3 of the population required a total gastrectomy. The postoperative morbidity was 25.9% and surgical mortality was 1.7%. These quality standards were within the parameters presented n the latest Pan American gastric cancer consensus in 2016.

The SVg5 of the patients in this study was 56.9%, with this number being comparable to others published in the literature^5^ ^,^ ^11^. 

With regards to the prognostic factors of long-term survival, guidelines have been developed with the objective of providing assistance for deciding which therapeutic strategies should be pursued. In this manner, TNM^15^ classification has allowed to guide the management of these patients.

The prognostic effectiveness of the TNM classification and its studies are well known and have been analyzed by different international^1^ ^,^ ^13^ ^,^ ^14^and domestic study groups^5,11,20.^

Regarding the N+/T index, it is reasonable to think that the lymph node metastatic potential of a tumor, depending on its level of invasion, could reliably predict patient prognosis. Thus, the ratio between compromised lymph nodes and T classification was devised, which was able to demonstrate significant differentiation between the global survival curves of the different subgroups (Figure 1) and different scenarios (Figures 2, 3 and 4). The only two curves that did not reach statistical significance were the comparison between N+/T subgroups B vs. C, in 1) the positive signet ring cell GC and 2) distal GC that received subtotal gastrectomy. One possible explanation for this finding is the small number of patients in each arm, another explanation for this phenomenon in the positive signet ring cell GC, is the peritoneal tropism which contributes in the worst prognosis, although the evidence in this point is contradictory^12^ ^,^ ^19^ ^,^ ^21^. This could possibly explain why the survival did not differ between the N+/T subgroups B and C. 

**FIGURE 5:**
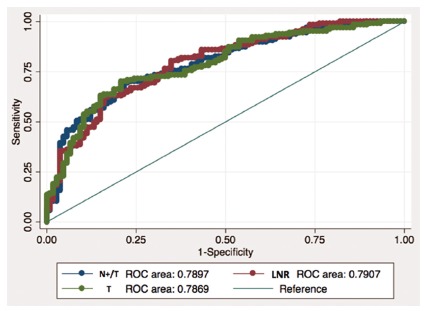
ROC curve of N+/T, T and LNR: areas under the curve are 0.78, 0.78 and 0.79 respectively

Furthermore, the analysis of ROC curves (Figure 5) demonstrates how the N+/T index has an area below the curve of 0.789, without a significant statistical difference of T and LNR factors (p=0.96) which are known to be well establish prognostic factors.

The strengths of this investigation are the following: 1) The study of a numerous population that represents the international and domestic reality; 2) said population has been managed following international treatment standards for gastric cancer with a curative intent by a reduced number of expert surgeons; 3) the provision of a new survival prediction index.

The weaknesses of this investigation are as follows: 1) It covers a period of time in which there was a change in TNM classification (6^th^ and 7^th^ edition) which impacted the classification and quite possibly the management of these patients; 2) it doesn`t include the adjuvant therapy used in the analysis, this limitation is due to the absence of chemotherapy scheme registration in more than 20% of patients, the information bias of this under registration, cannot make conclusion reliable in adjuvant therapy. This happens because some health provisional system of patients in our hospital, can mandate an externalization of the service to another institution. 

## CONCLUSION

The N+/T index is a reliable quotient in the prognostic evaluation of gastric adenocarcinoma patients who have been resected with curative intent.
